# Use of Cetuximab in Combination with Cisplatin and Adjuvant Pelvic Radiation for Stage IIIB Vulvar Carcinoma

**DOI:** 10.1155/2015/139817

**Published:** 2015-07-29

**Authors:** Jennifer Bergstrom, Michael Bidus, Edward Miles, Jay Allard

**Affiliations:** Naval Medical Center Portsmouth, 622 John Paul Jones Circle, Portsmouth, VA 23708, USA

## Abstract

Vulvar cancer is a rare carcinoma constituting only 4% of gynecologic malignancies and 0.6% of female cancers. Most chemotherapy regimens have been created from extrapolation from anal and cervical cancer research. Advanced stages have the worst prognosis and oftentimes invasive surgical procedures are needed to cure disease with high recurrence rates. *Case.* A 50 yo G2P2 presented for a 2 cm mass in her right labia. The patient underwent a partial radical vulvectomy and bilateral superficial and deep inguinal lymph node dissection. Bilateral inguinal lymph nodes were positive for residual disease. The patient underwent whole pelvic radiation with cisplatin as a radiosensitizer. The primary tumor was epidermal growth factor receptor (EGFR) positive and cetuximab, a monoclonal antibody to EGFR, was added. The patient underwent seven cycles of chemotherapy including cisplatin and cetuximab with adjuvant radiation therapy to the pelvis. She currently is without evidence of disease recurrence since completing treatment 4 years ago. *Conclusion.* One previous case report showed short-term palliative success of five months for recurrent, metastatic vulvar cancer. This case suggests that cetuximab could possibly be used in initial management of patients with advanced stages of vulvar cancer to improve prognosis.

## 1. Introduction/Background

Vulvar carcinoma is a rare cancer compromising 4% of all gynecologic cancers and 0.6% of all female cancers. Incidence increases steadily with age and usually peaks in the 7th decade. The American Cancer Society has estimated for 2014 that there will be 4850 new cases of vulvar cancer diagnosed and 1030 women will die from the disease [1, 2].

Symptoms of vulvar cancer are usually pain, irritation, pruritis, bleeding, ulceration, and occasionally a visible lesion. Biopsy of any suspicious lesion is required for diagnosis. Treatment for vulvar cancer typically involves surgery with or without adjuvant radiation therapy and/or chemotherapy [2].

The prognosis for vulvar cancer is generally good but dependent upon stage. Five-year overall survival rate for stages I and II vulvar carcinoma is 80–90%. Once a patient develops stage III disease, survival drops to 48%. With stage IV disease, 5-year overall survival is 15%. Lymph node involvement is the single most important prognostic factor. The presence of inguinal lymph node metastasis reduces survival by up to 50% [2].

There is no standard chemotherapy regimen for vulvar cancer. Extrapolation from regimens used to treat anal and cervical carcinomas has guided current treatment algorithms. Chemotherapy regimens have included 5-fluorouracil (5-FU), cisplatin, mitomycin-C, and bleomycin [3].

Cetuximab is a recombinant, chimeric monoclonal antibody that is directed against the epidermal growth factor receptor (EGFR) and has been shown to have antineoplastic activity. This biologic agent binds to the extracellular domain of the EGFR and prevents the activation and dimerization of the receptor. The antagonist activity at the receptor may result in inhibition in signal transduction and antiproliferative effects. Cetuximab may inhibit EGFR-dependent primary tumor growth and metastasis. It is currently being used in advanced and recurrent colorectal and head and neck cancers [4].

We present a case of advanced stage vulvar cancer with metastasis to inguinal lymph nodes and positive margins after surgical therapy that was treated with subsequent pelvic radiation and cisplatin with the addition of cetuximab.

## 2. Case

The patient is a 50 yo G2P2 who initially presented to her gynecologist for a 2 cm mass in her right labia, after noting bleeding with any sort of Valsalva-type maneuver such as defecating or urinating. She denied vaginal discharge, dyspareunia, or pain associated with the mass. She noted no change in bowel habits, dysuria, hematuria, hematochezia, melena, neurologic symptoms, or bone pain.

The patient had previously been scheduled for a dilation and curettage for menorrhagia by her gynecologist with simultaneous resection of the vulvar lesion. The labial lesion was initially thought to be a Bartholin's gland cyst.

Pathology from the dilation and curettage demonstrated benign endometrial tissue, but the vulvar excision revealed 1.5 × 1.7 × 0.7 cm mass that was consistent with moderately differentiated squamous cell carcinoma with basaloid features that extended to the edges of the biopsy. The pathology was reviewed at Johns Hopkins University where it was interpreted to be poorly differentiated carcinoma with squamous features that could have originated from an introital ductal structure such as a Bartholin gland duct or any other mucosal/skin adnexal structure. However, the pathologic differential diagnosis also included the possibility that the mass could be a metastatic tumor originating elsewhere in the anogenital tract.

The patient was referred to the Gynecologic Oncology Service where she was evaluated and underwent a position emission tomography/computed tomography scan (PET/CT) which demonstrated a thyroid lesion (later biopsied and found to represent a benign Hurthle cell lesion); mediastinal and bilateral hilar adenopathy (bronchoscopy with biopsy revealed benign disease consistent with sarcoidosis); a metabolically active right inguinal lymph node measuring 1.9 × 1.1 cm; and abnormal metabolic activity within the right vulvar region (see Figure 2).

A partial radical vulvectomy with bilateral superficial and deep inguinal lymph node dissection was performed for newly diagnosed squamous cell carcinoma of the vulva. Despite what was clinically noted to be an adequate area of dissection (excision 2.5 × 2.0 cm and depth of 3.0 cm), pathology demonstrated residual poorly differentiated carcinoma with squamous features measuring 0.6 cm with positive surgical margins along the deep margin. A right inguinal lymph node measuring 2.0 cm demonstrated metastatic disease. Two of ten left inguinal lymph nodes were positive for metastatic disease, with the largest measuring 0.7 cm.

**Figure 1:**
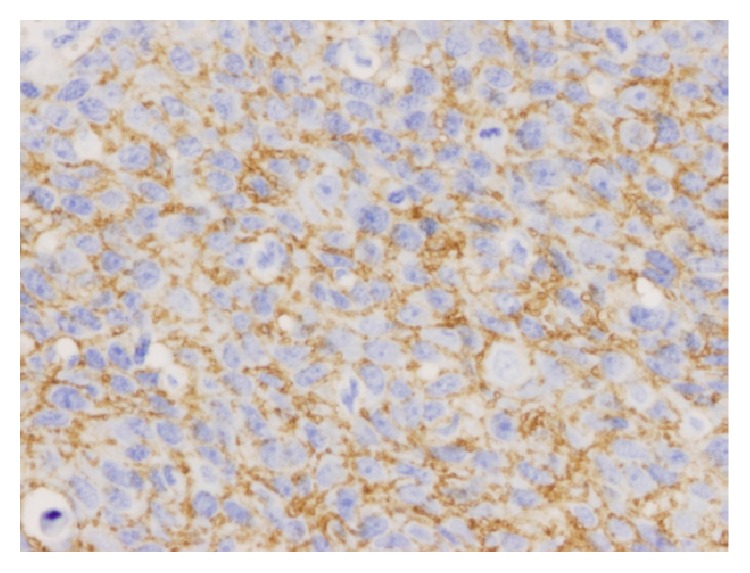
EGFR immunostain with cytoplasmic staining. The brown staining shows where the EGFR is detected.

Our patient was determined to have FIGO stage IIIB squamous cell carcinoma of the vulva (see Figure 1). Due to positive surgical margins and positive inguinal lymph nodes, our patient required full pelvic and inguinal-femoral radiation and chemotherapy with cisplatin as a radiosensitizer. Given our concern for the high risk of recurrence, the patient's primary tumor was tested and noted to be epidermal growth factor receptor (EGFR) positive (see Figure 1). The decision was made to add cetuximab to her chemotherapy regimen. She underwent weekly cisplatin therapy dosed at 40 mg/m^2^ with the addition of cetuximab dosed at 400 mg/m^2^ for seven cycles along with adjuvant radiation therapy to the pelvis. Radiation therapy was dosed in the standard fashion at 4,500 cGy at 180 cGy/fraction for a duration of 25 fractions in 34 days followed by 1,440 cGy at 180 cGy/fraction for 8 fractions over 9 days. The radiation treatment area included the low pelvis, inguinal lymph nodes, and vulva.

**Figure 2:**
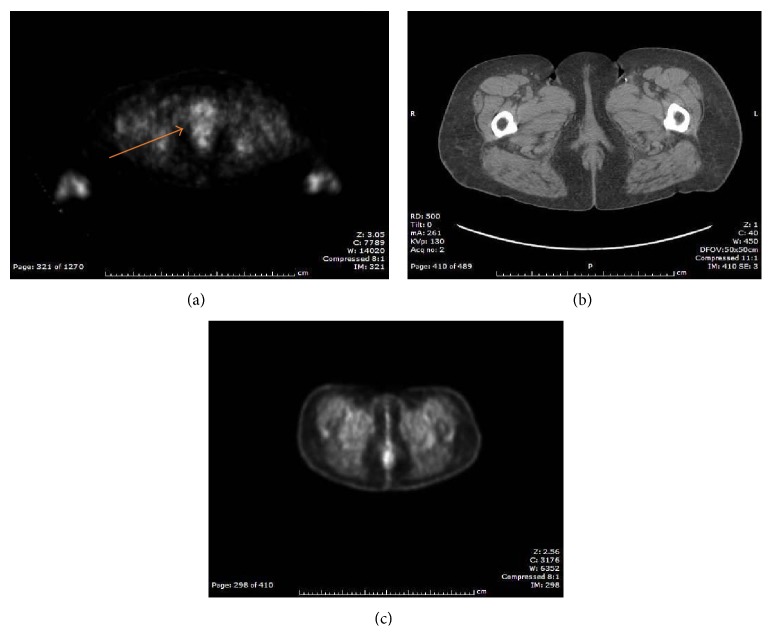
(a) PET CT before treatment. Arrow shows metabolically active area referring to vulvar lesion. (b) CT correlation. (c) PET CT after treatment.

The patient's postradiation and chemotherapy course was complicated by three hospitalizations for erysipelas. Once treated, the patient was prescribed once daily penicillin for prophylactic therapy. She also reported intermittent diarrhea alternating with constipation that was managed with diet and Miralax. She is currently being followed by our service on a regular basis and is without evidence of disease recurrence over 4 years after primary treatment (see Figure 2). She has undergone hyperbaric oxygen therapy due to radiation fibrosis and vesicular lesions on the vulva. She has had an excellent result with this treatment.

## 3. Discussion/Conclusion 

There is no standard chemotherapy regimen for vulvar cancer and much of the data that is being used to guide treatment is extrapolated from studies for cervical and anal cancer [3]. Advanced stages of vulvar cancer have a worse prognosis and invasive surgical procedures are often required to cure disease with high recurrence rates.

Epidermal growth factor receptor is within a family of receptors known as type I receptor kinases or ErbB receptors. The receptor family consists of ErbB1/EGFR/HER1, ErbB2/HER2, ErbB3/HER3, and ErbB4/HER4. ErbB receptors contain an extracellular ligand-binding domain, a transmembrane segment, and an intracellular protein tyrosine kinase domain that contains a regulatory carboxyl terminal segment. Activation of these receptors leads to downstream cell proliferation, survival, and transformation. If these receptors are deregulated it can lead to malignant transformation [5].

Cetuximab is a recombinant humanized monoclonal antibody that is targeted against the ligand-binding domain of the epidermal growth factor receptor. It competitively inhibits growth factor binding and inhibits autophosphorylation and cell signaling. It has been found that cetuximab enhances cytotoxic effects of radiation when used to treat squamous cell carcinoma [6]. In addition, it has been thought that cetuximab induces antibody mediated receptor dimerization which results in downregulation. This could affect growth inhibition [5]. In a series of phase I/II studies, cetuximab was found to be safe, with the most prominent side effects being an acneiform skin rash and anaphylactoid or anaphylactic reactions. These were noted to occur in approximately 2% of patients [5].

Johnson et al. found that there was increasing expression of EGFR in vulvar tissue that was malignant. EGFR was also significantly associated with lymph node metastasis and decreased patient survival [7]. There is some data from literature regarding squamous cell carcinoma of the head and neck, which often expresses increased EGFR, that suggests that the addition of cetuximab to standard chemotherapy and radiation regimens may cause improvement in overall survival, as well as progression-free survival [8, 9]. One study randomized patients with recurrent or metastatic squamous cell carcinoma to receive platinum-based chemotherapy plus fluorouracil or cetuximab plus platinum-fluorouracil chemotherapy [9]. Conclusions from this study showed the addition of cetuximab to the regimen improved overall survival when given as first-line treatment [9]. Another study that compared radiation therapy alone versus radiation therapy with the addition of cetuximab in the treatment of locoregionally advanced squamous cell carcinoma of the head and neck showed positive results [8]. Investigators saw a median duration of locoregional control of 24.4 months in the arm treated with radiation plus cetuximab compared to 14.9 months with those patients only given radiation. The overall survival was 49 months versus 29.3 months [8]. This was statistically significant and, arguably, clinically meaningful.

Vulvar carcinoma is a rare cancer that large, randomized trials, as noted above for head and neck cancer, would be difficult to perform. Therefore, extrapolation from other carcinomas with similar pathogenesis, such as head and neck cancer, can be imperative to new treatment regimens for vulvar carcinoma.

In this case report cetuximab was added to cisplatin chemotherapy regimen given the primary tumor was EGFR positive. One previous case report showed short-term palliative success of 5 months for recurrent, metastatic vulvar cancer [10]. This case report shows the promise that cetuximab could hold for advanced stage vulvar cancer that is EGFR positive during first-line treatment.
